# Determining the Optimum Power Load in Jump Squat Using the Mean Propulsive Velocity

**DOI:** 10.1371/journal.pone.0140102

**Published:** 2015-10-07

**Authors:** Irineu Loturco, Fabio Yuzo Nakamura, Valmor Tricoli, Ronaldo Kobal, Cesar Cavinato Cal Abad, Katia Kitamura, Carlos Ugrinowitsch, Saulo Gil, Lucas Adriano Pereira, Juan José González-Badillo

**Affiliations:** 1 NAR - Nucleus of High Performance in Sport, São Paulo, SP, Brazil; 2 Faculty of Sport, Pablo de Olavide University, Seville, Spain; 3 State University of Londrina, Londrina, PR, Brazil; 4 School of Physical Education and Sport, University of São Paulo, São Paulo, SP, Brazil; West Virginia University School of Medicine, UNITED STATES

## Abstract

The jump squat is one of the exercises most frequently used to improve lower body power production, which influences sports performance. However, the traditional determination of the specific workload at which power production is maximized (i.e., optimum power load) is time-consuming and requires one-repetition maximum tests. Therefore, the aim of this study was to verify whether elite athletes from different sports would produce maximum mean propulsive power values at a narrow range of mean propulsive velocities, resulting in similar jump heights. One hundred and nine elite athletes from several individual/team sport disciplines underwent repetitions at maximal velocity with progressive loads, starting at 40% of their body mass with increments of 10% to determine the individual optimum power zone. Results indicated that regardless of sport discipline, the athletes’ optimum mean propulsive power was achieved at a mean propulsive velocity close to 1.0 m.s^−1^ (1.01 ± 0.07 m.s^−1^) and at a jump height close to 20 cm (20.47 ± 1.42 cm). Data were narrowly scattered around these values. Therefore, jump squat optimum power load can be determined simply by means of mean propulsive velocity or jump height determination in training/testing settings, allowing it to be implemented quickly in strength/power training.

## Introduction

Muscle power ability is probably the most important aspect for determining performance in a range of different sports such as rugby, martial arts, soccer and track and field [1]. Most actions involved in specific technical and locomotor activities are dependent on the capacity to apply a significant amount of force at high speed. Indeed, research has demonstrated strong correlations between power ability and specific sport performance [2–5]. For instance, the jump squat power production presents a very large positive correlation with punching acceleration in karate [4]. Additionally, sprinters with higher values of lower limb muscle power perform better in speed tests [2,3]. The same holds true for rugby players, for whom power outputs and rate of force development in the deadlift and jump squat can be used to predict performance in field sprint, vertical jump and change-of-direction tests [5].

For this reason, strength and conditioning coaches (SCC) have sought training methods capable of improving athletes’ power production capacity. These training methods are usually based on very specific exercises and workloads. In this regard, the jump squat is one of the exercises most frequently used to achieve such a purpose, since it is able to improve both lower extremity power and specific sport physical performances [6,7]. Although many authors recommend a “mixed-model” training strategy to increase muscle power [8–10], there seems to be a specific workload—generally expressed as a percentage of the one-repetition maximum (1RM)—at which power production is maximized (i.e., optimum power load), and, consequently, training effects optimized [1,6,11,12].

Determination of the jump squat optimum power load is somewhat complex and time-consuming, which complicates the training load adjustments throughout a training cycle. Initially, the squat 1RM load for each athlete is determined. Following this, the athletes usually perform several sets at a wide range of percentages of the 1RM (i.e., 0–90%) to determine the exercise-load that maximizes power production [8,13,14]. This procedure is necessary because there are reports of the jump squat optimum loads ranging from zero to 90% of the 1RM load [13–15].

Under this perspective, methods capable of reducing the number of attempts and testing duration can be very useful in terms of time, fatigue and safety. One alternative would be using a method that does not require determination of the 1RM. For instance, Marques et al. [16] used three fixed loads (i.e., 26, 36, and 46 kg) to determine the power production and bar-velocity in the bench press, executed by a group of elite handball players. However, using fixed loads may not be feasible when evaluating elite athletes from different sports and training backgrounds (i.e., long jumpers vs. endurance runners). Conversely, the possibility of fixing percentages of body mass as a load parameter may be interesting [17], as this does not require 1RM testing, respecting the neuromuscular/anthropometric characteristics of the subjects [18]. Nevertheless, the development of a multifaceted approach incorporating (in addition to the “load parameter”) the force-velocity relationship would be very useful to assess athletes and to propose new strength-power training strategies [8].

Power production is enhanced when one or both components of the power equation (*i*.*e*., *Power* = *Force* × *Velocity*) are enhanced. However, it is interesting to note that some studies have reported that the maximum power production is achieved at velocities close to 1 m·s^−1^ both in the bench press [19] and squat [15] exercises. However, the load capable of maximizing the power output is dependent on the method used to find this variable [18]. Therefore, it is necessary to develop a more appropriate method to determine the variable (i.e., mean propulsive power [MPP]) which better represents the optimum power load.

For instance, mean power and peak power collected during the upward portion of the movement are widely used to assess sport performance and to optimize training strategies [7,12]. Mean power is calculated as the respective area under the upward portion of the power-time curve, while peak power represents the maximum instantaneous value obtained during a given upward movement [20]. Despite their extensive use as reference values of muscle power, the varied spectrum of loads used to assess these variables produce a large dissimilarity in the outputs obtained [1,21].

Recently, MPP has been suggested for assessing muscle power, since this parameter may avoid underestimating an individual’s neuromuscular potential when lifting light or medium loads [22]. While mean power refers to the entire upward portion of a lift (including the braking phase, when a < -g), MPP refers to the portion of the upward movement during which the measured acceleration is greater than the acceleration related to gravity (i.e., a ≥ 9.81 m·s^−2^). This consideration is valid for both traditional exercises (where the deceleration occurs due to the necessity “to brake” at the end of the range of motion) and ballistic jumps (where the deceleration during the upward phase occurs after the take-off). Thus, powerful athletes should accelerate more rapidly during the upward movements of the jumps, diminishing the relative time of the accelerating phase (i.e., time spent by a given athlete applying force against the ground) and increasing the relative time of the decelerating phase (i.e., time-interval between the beginning of the take-off and the “zero-velocity”, attained immediately before the commencement of the downward phase). Based on this mechanical principle, the outcomes related to the entire upward portion of a given movement (i.e., mean power values) may be biased. In addition, a training system based on MPP is capable of enhancing performance at both ends of the force-velocity curve, producing significant improvements both in sprinting and jumping ability, and maximum dynamic strength (squat 1RM) [11].

Due to the potential importance of MPP to assess and train elite athletes, it is essential to find a more accurate and time-saving method able to identify this variable. Since the maximum power production in bench press and squat exercises occurs at the same velocity [15,19], we hypothesized that the same would hold true for the jump squat exercise, even using the MPP as a reference value (instead of peak power or mean power). Therefore, the purpose of this study was to verify whether elite athletes from different team and individual sport disciplines produce their jump squat maximum MPP at a narrow range of mean propulsive velocities, which would allow the use of a fixed velocity value to determine the optimum power load.

## Methods

### Study Design

**Fig 1:**
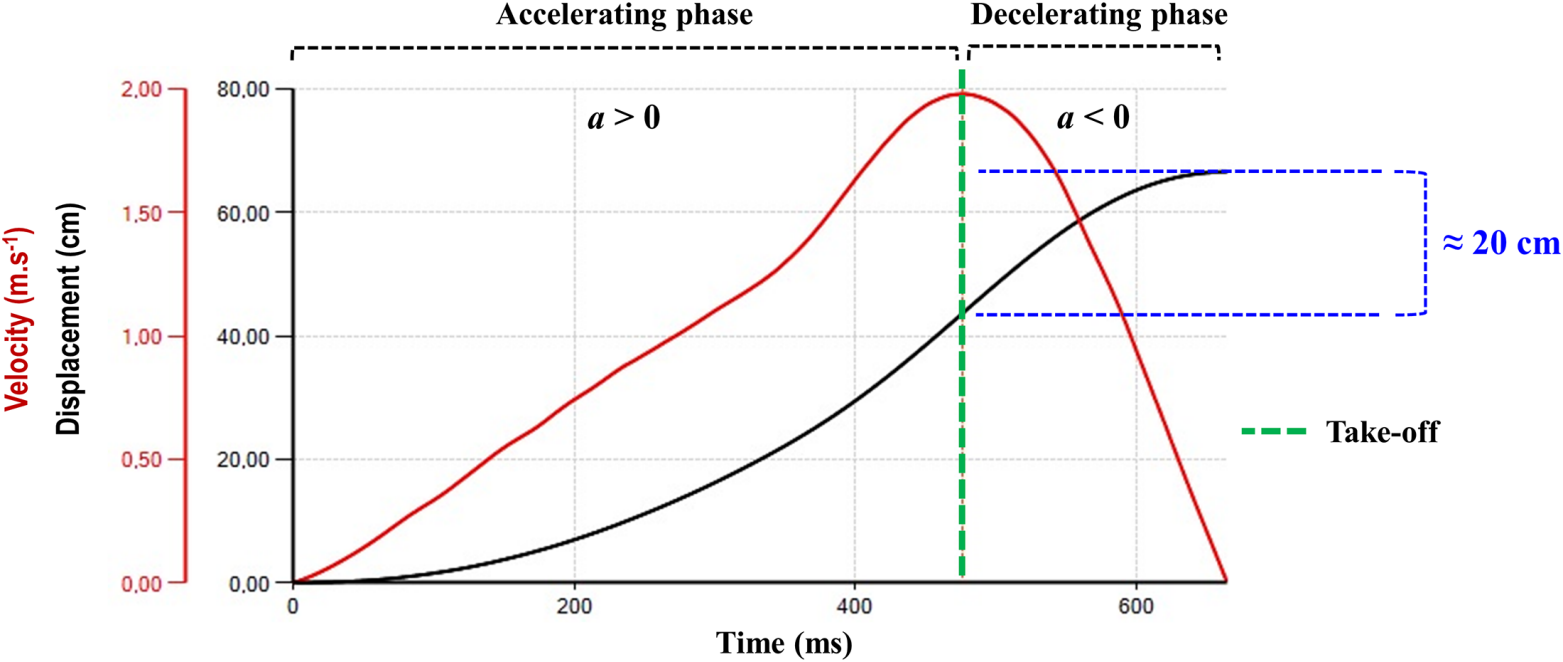
Accelerating and decelerating phases during an actual jump squat attempt. The measurement was performed at the optimum power zone. From the take-off (when the velocity begins to decrease) to the final point of the upward movement (at “zero-velocity”) the athlete vertically jumps ≈ 20 cm.

All elite athletes involved in this cross-sectional investigation were assessed during the competitive phase of the season, after being familiarized by performing (minimum) six power training sessions, using the same exercise (jump squat), equipment (Smith machine, encoder and contact platform) and experimental procedures as the actual assessment. The subjects were required to refrain from any heavy workout for 3 days prior to the testing day, fast for 2 h before attending the session and avoid caffeine and alcohol consumption for 24 h before the study. As the bar-velocity is the central premise of this methodological research, before each jump squat attempt, the athletes were strongly encouraged to move the bar upward rapidly, jumping as fast and as high as possible. The variables analyzed is this study were: *(1)* MPP—mean value that refers to the upward portion of the jump squat during which bar-acceleration is greater than acceleration due to gravity; *(2)* mean propulsive velocity (MPV)—velocity measure which corresponds to the mean velocity of the propulsive phase of each repetition; and *(3)* jump squat height—the height reached by the athlete (the rise of the center of gravity above the ground) when performing jump squats. For the sake of clarity, Fig 1 shows the accelerating and decelerating phases that occur during the upward portion of a jump squat exercise performed at the optimum power zone.

### Subjects

One hundred and nine elite athletes from six sport disciplines (allocated into seven groups) volunteered to participate in this study: power track & field athletes ([P T&F] sprinters, jumpers, throwers (javelin and shot put), decathletes and heptathletes; men: n = 30; women: n = 20), rugby and American football players ([RB/AF] men: n = 9), soccer players (men: n = 19), endurance runners (men: n = 11), combat sports athletes (karate and taekwondo; men: n = 12), and tennis players (men: n = 8). The characteristics of each group are shown in Table 1. The sample comprised athletes involved in national and international competitions, including members of Brazilian national teams, Olympic, Pan-American and National medalists, and World Champions, thus attesting to their high level of competitiveness. Among the tennis players, specifically, there were Grand Slam and/or Davis Cup competitors. Subjects were briefed on the experimental risks and benefits of the research, and signed an informed consent form agreeing to take part. The study was approved by the University of São Paulo, School of Physical Education and Sport Ethics Committee, and conformed to the Declaration of Helsinki.

**Table 1:** Characteristics of the subjects in the elite individual/team sport athletes.

	Age (years)	Height (cm)	Body Mass (kg)
**P T&F Men**	24.0 ± 5.2	179.0 ± 5.9	77.9 ± 8.5
**P T&F Women**	23.2 ± 4.4	169.0 ± 6.9	60.6 ± 10.6
**RB/AF**	22.0 ± 2.9	183.2 ± 5.3	104.6 ± 21.1
**Soccer**	24.2 ± 4.1	177.0 ± 6.9	71.9 ± 6.8
**Endurance Runners**	24.7 ± 4.3	173.2 ± 3.1	62.3 ± 2.7
**Combat Sports**	21.9 ± 2.4	173.0 ± 4.8	66.2 ± 8.9
**Tennis**	22.7 ± 5.4	180.0 ± 5.2	77.9 ± 6.1

*Note*: P T&F = power track & field (sprinters, jumpers, throwers, decathletes, and heptathletes); RB/AF = rugby and American football; Combat Sports = karate and taekwondo.

### Testing Day

After a standardized 15-minute warm-up including general (i.e., running at a moderate self-selected pace (rating of perceived exertion corresponding to 4, in the CR10 Borg scale [23]) for 5 minutes followed by 5 minutes of lower limb active stretching) and specific exercises (i.e., plyometrics and jump squats using light-loads for 5 minutes), subjects were provided with a 5-minute rest interval. Following this, the athletes were required to execute the mean propulsive power tests.

### Assessment of mean propulsive power, mean propulsive velocity and jump squat height at the optimum power load

MPP, MPV, and jump squat height were assessed in the jump squat exercise, performed on a Smith machine (Technogym Equipment, Italy). The elite athletes were instructed to execute 3 repetitions at maximal velocity for each load, starting at 40% of their body mass. Subjects executed a knee flexion until the thigh was parallel to the ground and, after an initial command, jumped as fast as possible. Prior to each muscle power assessment, an experienced test administrator instructed the participant to maintain constant downward pressure on the barbell throughout the jump, to prevent the bar moving independently of the body [18,24]. A load of 10% of body mass was gradually added in each set until a decrease in MPP was observed. This was observed after, on average, 5–6 sets. A 5-minute interval was provided between sets. To determine MPP and MPV, a linear transducer (T-Force, Dynamic Measurement System; Ergotech Consulting S.L., Murcia, Spain) was attached to the bar on the Smith machine. The finite differentiation technique was used to calculate bar velocity and acceleration, presenting an associated error of < 0.25%, while displacement was accurate to ± 0.5 mm [22]. Instantaneous bar velocity was sampled at a frequency of 1000 Hz. A digital filter with no phase shift was subsequently applied to the data. The resolution of the A/D card is 14 bits. The displacement was obtained by integration of v data with respect to time; instantaneous acceleration (a) was obtained from differentiation of v with respect to time; instantaneous force (F) was calculated as F = m · (a + g), where m is the moving mass (kg) and g is the acceleration due to gravity [22,25,26]. The proprietary software used is commercial and was provided by the manufacturer in conjunction with the equipment. The jump squat height was determined using a contact platform (Smart Jump; Fusion Sport, Coopers Plains, Australia), being estimated by the flight time, according to the standard procedures reported by Bosco et al. [27]. We considered the MPP, MPV and jump squat height collected at the optimum power zone (maximum value of mean propulsive power) for data analysis purposes. In order to avoid misinterpretation of the power outputs and taking into considering the influence of body mass on its calculation and, consequently, on the bar-velocity and squat jumping height, we normalized these values by dividing the absolute power value by the body mass (MPP REL, relative power = W/kg). Test-retest reliability for jump squat outputs as measured by the coefficient of variation was < 5%.

### Statistical Analysis

Normality was checked via the Shapiro Wilk test. The means, standard deviation (SD) and confidence interval (CI 95%) were used to represent centrality and spread of data. A One-Way ANOVA with Hochberg's GT2 post-hoc analysis was used to compare MPP, MPP load, the maximum MPP load expressed as a percentage of body mass (MPP_BM_), MPP REL, MPV, and jump height among the seven groups of elite athletes. The effect sizes (ES; Cohen’s d [28]) of significant differences detected by ANOVA were calculated among the groups. The significance level was set at *P* < 0.05.

## Results

The absolute MPP, MPP load, MPP_BM_, MPP REL, MPV and jump height in the jump squat exercise for the seven groups of individual and team sports athletes are presented in Table 2. The absolute MPP was higher in the P T&F men (ES = 2.68, 3.40, 4.78, 3.32, and 3.75compared to P T&F women, soccer, endurance runners, combat sports, and tennis, respectively; P < 0.05) and RB/AF (ES = 1.64, 2.28, 3.58, 2.23, and 2.62, compared to P T&F women, soccer, endurance runners, combat sports, and tennis, respectively; P < 0.05) than in the other groups. The soccer players presented higher absolute MPP than the endurance runners (ES = 1.45; P < 0.05) (Table 2).

**Table 2:** Mean propulsive power (MPP), MPP load (kg), optimum MPP load expressed as percentage of body mass (MPP_BM_), relative MPP (MPP REL), mean propulsive velocity (MPV), and jump height performance in the elite individual/team sport athletes.

	MPP (W)	MPP Load (kg)	MPP_BM_ (%BM)	MPP REL (W·kg^−1^)	MPV (m·s^−1^)	Jump Height (cm)
**P T&F Men**	1117.7 ± 129.3	80.6 ± 10.6	103.4 ± 7.1	14.40 ± 1.47	1.03 ± 0.05	21.33 ± 1.49
**P T&F Women**	778.6 ± 124^#^ ^¥^	60.4 ± 9.9^#^ ^¥^ ^¶^	100.0 ± 8.2	12.95 ± 1.71	0.98 ± 0.06	19.85 ± 1.04
**RB/AF**	997.2 ± 142.9	79.7 ± 17.5	77.2 ± 14.5^#^ *	9.74 ± 1.7^#^ *	0.99 ± 0.04	21.08 ± 1.22
**Soccer**	705.5 ± 113.5^#^ ^¥^ ^¶^	54.3 ± 9.2^#^ ^¥^	76.0 ± 14.6^#^ *	9.92 ± 2.09^#^ *	1.00 ± 0.07	20.39 ± 1.73
**Endurance Runners**	543.0 ± 111.1^#^ ^¥^	41.6 ± 7.7^#^ ^¥^	66.5 ± 10.3^#^ * ^§^	8.68 ± 1.52^#^ *	1.00 ± 0.04	19.73 ± 0.71
**Combat Sports**	704.0 ± 120.2^#^ ^¥^	54.4 ± 11.1^#^ ^¥^	82.0 ± 11.4^#^ *	10.64 ± 1.15^#^ *	1.00 ± 0.05	20.10 ± 1.47
**Tennis**	659.3 ± 115.4^#^ ^¥^	54.2 ± 8.6^#^ ^¥^	69.6 ± 9.2^#^ *	8.46 ± 1.25^#^ *	0.95 ± 0.04	20.27 ± 1.31

*Note*: P T&F = power track & field (sprinters, jumpers, throwers, decathletes, and heptathletes); RB/AF = rugby and American football; Combat Sports = karate and taekwondo.

^#^Different from Power Track & Field Men;

*Different from Power Track & Field Women;

^¥^Different from RB/AF;

^¶^Different from Endurance Runners;

^§^Different from Combat Sports (*P* < 0.05).

The MPP load was greater in the P T&F men (ES = 1.97, 2.66, 4.27, 2.42, and 2.75 compared to P T&F women, soccer, endurance runners, combat sports, and tennis, respectively; P < 0.05) and RB/AF (ES = 1.41, 1.91, 3.03, 1.78, and 1.95, compared to P T&F women, soccer, endurance runners, combat sports, and tennis, respectively; P < 0.05) than in the other groups. The P T&F women demonstrated higher MPP load when compared to the endurance runners (ES = 2.13; P < 0.05) (Table 2).

Both men and women P T&F athletes attained their maximum MPP at higher percentages of body mass than the remaining groups of athletes (men [ES = 2.43, 2.52, 4.23, 2.32, and 4.15 compared to RB/AF, soccer, endurance runners, combat sports and tennis groups, respectively; P < 0.05] and women [ES = 2.00, 2.10, 3.60, 1.83, and 3.49compared to RB/AF, soccer, endurance runners, combat sports and tennis groups, respectively; P < 0.05]). The combat sports athletes showed higher percentage of optimum MPP load expressed as percentage of body mass than the endurance runners (ES = 1.43; P < 0.05) (Table 2).

The MPP REL was higher in the P T&F men (men [ES = 2.94, 2.52, 3.83, 2.87, and 4.37 compared to RB/AF, soccer, endurance runners, combat sports and tennis groups, respectively; P < 0.05)] and women [ES = 1.88, 1.59, 2.64, 1.62, 3.03 compared to RB/AF, soccer, endurance runners, combat sports and tennis groups, respectively; P <0.05]) than in the other groups of athletes (Table 2). No differences were observed in the MPV or squat jump height among the groups.

**Fig 2:**
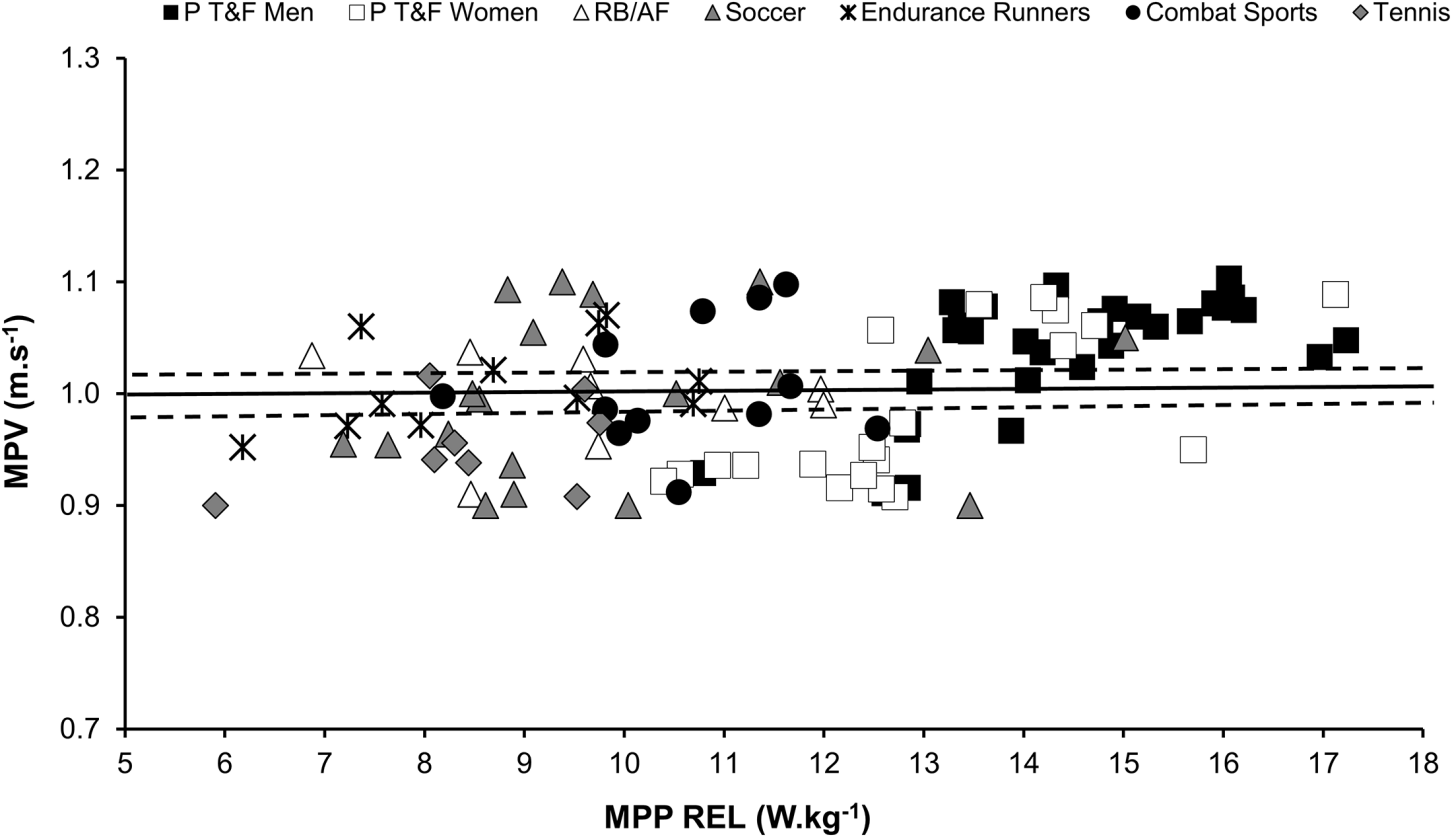
Mean propulsive velocity (MPV) and relative mean propulsive power (MPP REL) of elite individual/team sport athletes. The central, unbroken line represents the mean and the dashed lines represent the confidence interval (95%) of the MPV of all athletes. P T&F = power track & field (sprinters, jumpers, throwers, decathletes, and heptathletes); RB/AF = rugby and American football; Combat Sports = karate and taekwondo.

**Fig 3:**
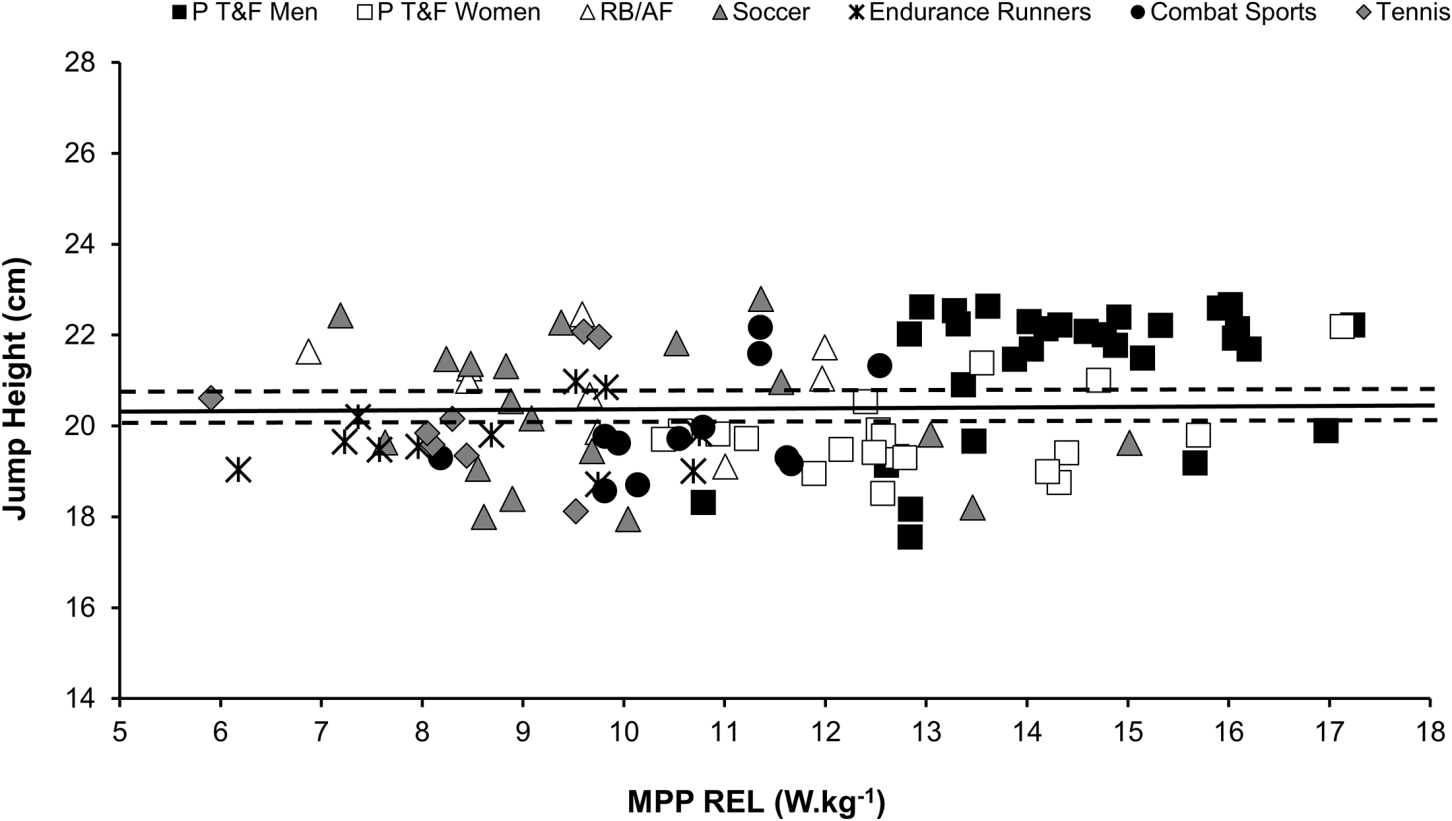
Jump height and relative mean propulsive power (MPP REL) of elite individual/team sport athletes. The central, unbroken line represents the mean and the dashed lines represent the confidence interval (95%) of the jump heights of all athletes. P T&F = power track & field (sprinters, jumpers, throwers, decathletes, and heptathletes); RB/AF = rugby and American football; Combat Sports = karate and taekwondo.

Fig 2 presents the MPV and MPP REL in the jump squat exercise for all athletes separated by the groups. The mean (± SD) of MPV for all athletes (analyzed together) was 1.01 ± 0.07 m.s^−1^(95% CI: 0.99, lower bound; 1.02, upper bound). In Fig 3 the jump height and MPP REL in the jump squat exercise are presented for all athletes separated in each group. The mean (± SD) of jump height for all athletes analyzed together was 20.47 ± 1.5 cm (95% CI: 20.19, lower bound; 20.74, upper bound).

## Discussion

This methodological report was designed to verify whether athletes from different sport disciplines would achieve their highest values of MPP in jump squats (i.e., optimum power load) at the same MPV. After examining the data collected from a sample that comprised 109 elite athletes who executed jump squats using different loads, we concluded that the higher values of MPP occur at ≈ 1 m·s^−1^. In addition, when these athletes move a bar at an MPV of ≈ 1 m·s^−1^ during a jump squat, they can reach a height of about 20 cm.

**Fig 4:**
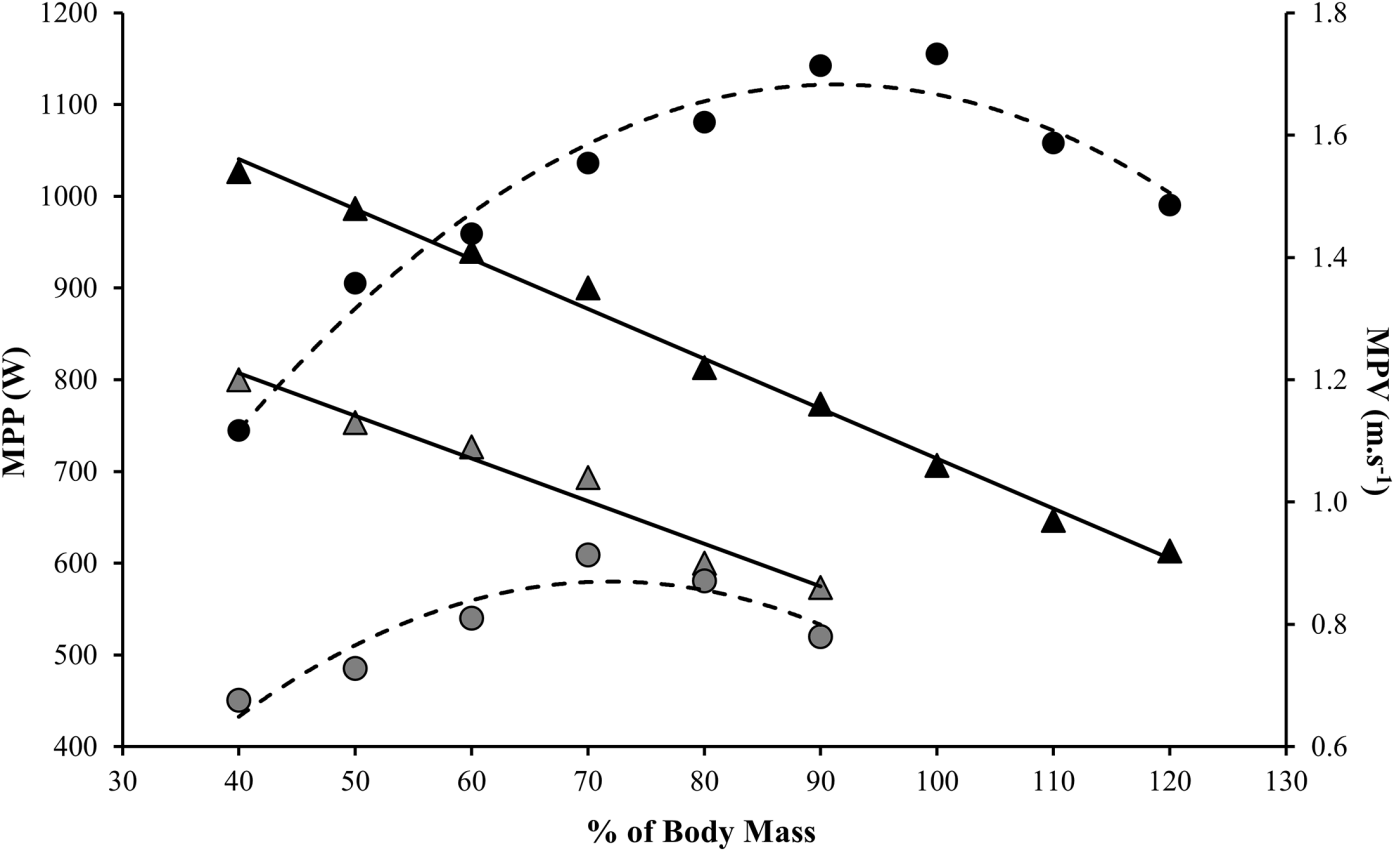
Relation between mean propulsive power (MPP) and mean propulsive velocity (MPV). The polynomial lines represent the MPP and the rectilinear lines represent the MPV. The presented values were obtained during actual testing attempts. Black symbols represent a power track and field athlete and the grey symbols represent an endurance runner. Independent of the power values, both athletes achieve the optimum power zone at 1 m·s^−1^.

The fact that elite athletes from different specialties and with different neuromuscular profiles (i.e., force-velocity [F-V] characteristics) can achieve their optimum values of MPP at the same MPV is an important novelty in sport science (as exemplified by Fig 4, which compares an endurance runner with a power track & field athlete). This finding is in accordance with Samozino et al. [29], who proposed that athletes with differing F-V characteristics would likely optimize power under different external loading conditions. It appears that at this bar-velocity (≈ 1 m·s^−1^)–for some reason that should be further clarified—the mean propulsive power is optimized, independent of the strength-power level of the subjects. Of note, in our study, the subjects with higher values of relative muscle power (P T&F group, *P* < 0.05) or with lower values of relative muscle power (team sports athletes, endurance runners, combat sports athletes and tennis players) obtained their maximum MPP results at around 1 m·s^−1^. Importantly, in spite of the optimum jump squat loads occurring at the same MPV (≈ 1 m·s^−1^), the different groups of athletes achieved their respective optimum MPP at distinct percentages of body mass (as expressed in Table 2). This enables coaches and sports scientists to considerably reduce the time spent assessing MPP, by starting the tests tailored by these reference loads or adjusting/increasing the load more sharply in the next set (rather than using 10% increment steps). Based on this measurement, sport scientists and SCC who use linear encoders and/or accelerometers to determine the optimum loads for their athletes may significantly reduce the time spent assessing power, and still achieve highly accurate test results. Moreover, this practical method avoids the “traditional power-training testing & prescription” [6,12], which is based on a range of 1RM percentages. This information is crucial for routine assessments in elite sports, since head coaches and fitness coaches tend to avoid using 1RM tests due to their time-consuming nature, volitional engagement and the inherent risks involved.

From a mechanical perspective, it is expected that individuals who jumped from a similar position/trajectory (vertical jump squats) and presented the same MPVs during a short push-off phase (≈ 450 ms) would reach similar take-off velocity when executing a vertical jump [30–32]. As the vertical jump height is determined by the take-off velocity and our subjects presented the same MPV during the jump squats under optimum power conditions (≈1 m·s^−1^), the athletes were able to jump a height of about 20 cm when using their individual optimum loads. It is important to emphasize that all power tests analyzed in this study were conducted with the athletes executing the jump squat repetitions as fast as possible. This methodological point is essential during maximum power assessments, since if a given subject moves a relative submaximal load at a submaximal velocity (in relation to the maximal velocity that could be applied against this relative load) the power value estimated will be inaccurate.

The most applicable and practical finding which emerges from this study is the possibility of monitoring the muscle power outputs during workouts using simple measures of vertical jump height in the jump squat exercise. This innovative possibility may be a useful and economical way to assess elite and recreational athletes. Although this method is applicable only for jump squats (performed under a perpendicular ground-trajectory), this new approach allows SCC to perform accurate muscle power tests using inexpensive contact platforms. Furthermore, it is possible to calculate the optimum load using the equation proposed by Bosco et al. [27], where the flight time (t) obtained for a subject is used to estimate the height of the body’s center of gravity (h) during a vertical jump attempt (i.e., h = g·t^2^/8; g = 9.81 m·s^−2^). The time variable required to complete this equation can be assessed using simple video analysis and the time spent jumping ≈ 20 cm in a vertical trajectory is around 400 milliseconds. Therefore, the assessment of muscle power using the height as a reference value should be conducted whilst increasing or reducing the jump squat load throughout the attempts, until the athletes achieve a height ≈ 20 cm (i.e., or a flight time around 400 ms). Importantly, since the optimum loads occur at narrow ranges of MPV (≈ from 0.9 to 1.1 m·s^−1^) and heights (≈ from 18 to 22 cm), the athlete can slightly vary his/her training velocity during a muscle power-oriented set, without leaving the “optimum power zone”, avoiding fatigue effects [25] or load increases due to the potentiation effect [33]. These possibilities are of practical interest, since SCC can implement optimum power loading in their training routines using different kinematic methods.

Finally, since linear position transducers (i.e., encoders) are widely used by coaches, the prospective opportunity to execute jump squats in the optimum power zone during all training sessions without performing any previous assessments (by simply adjusting the training load within the proper training session according to the bar-velocity) is a key advance in improving elite athletes’ performance. It has been widely established that *optimum-load jump squats* are strongly correlated with a series of sport-specific movements and functional performance tests [34,35]. Furthermore, training at the optimum power zone may produce similar performance enhancements to “traditional strength training”, promoting significant improvements at both ends of the force-velocity curve (i.e., high-force/low-velocity zone and low-force/high-velocity zone) [11].

We are aware that many authors refer to optimum power loads by using the peak power instead of the mean values of power [1,8]. However, it is important to emphasize that the MPP has been shown to be extensively associated with specific sports performance (i.e., sprinting, jumping and punching) [4,36,37]. Indeed, training at the optimum propulsive power zone produced similar performance improvements to traditional strength training [11] and is capable of reducing the power and speed decrements, which commonly occur during team sports preseasons [38]. However, we acknowledge the limitation of using only the jump squat exercise in this study. Whilst the jump squat is a widely used exercise to develop strength-power characteristics in athletes [39–41], determining the MPV associated with optimum loads in other exercises (e.g., bench press and bench throw) would benefit SSC in training athletes to perform functional tasks with the upper extremities, such as throwing and punching (e.g., handball ball throwing and boxing strokes). Secondly, another limitation that emerges from this study is the lack of substantial information regarding the mechanisms involved in the similarity of MPV at the optimum power load among groups differing in strength-power capacities. Nevertheless, it seems that at an MPV of 1.0 m·s^−1^ the relation between the overload and the bar-velocity in the JS exercise acts by maximizing the athletes’ muscle power production, regardless of their distinct inherent characteristics and training backgrounds (e.g., muscle fiber composition and sport specialty).

In summary, our data confirmed that athletes with different neuromuscular characteristics obtain their maximum values of MPP in jump squat exercises at the same MPV (≈ 1 m·s^−1^). Consequently, when performing *optimum-load jump squats*, they reach a height of about 20 cm. Based on these specific values of bar-velocity and vertical jump height, it is possible to reduce the time spent and increase the practicality of determining the maximum values of MPP. Because using optimum power load is capable of improving specific performance in a wide range of sport disciplines, SCC are encouraged to develop strength-power programs utilizing this novel scientific methodological approach. Further research is necessary to ascertain the mechanisms explaining why the optimum power load is found at similar MPV values across the different groups of athletes.

## Conclusion

Measuring the maximum power output is a common practice in sport science. Researchers and fitness coaches generally use optimum power loads to assess and train elite athletes, in order to improve their athleticism. Findings from this study substantially reduce the time required to test the MPP in elite athletes, and are in accordance with recent calls for sport science investigations aiming at valuable, simpler and timesaving methods, in order to bridge the gap between science and practice [42]. Importantly, from this point on, it is possible to determine the athletes’ optimum training zones, even in the absence of kinematic devices (i.e., linear encoders) by using the jump squatting height. This novel approach may provide a quick and reliable way to measure, monitor and train top-level athletes and strength-power trained subjects.
